# Wired for Intensity: The Neuropsychological Dynamics of Borderline Personality Disorders—An Integrative Review

**DOI:** 10.3390/jcm14144973

**Published:** 2025-07-14

**Authors:** Eleni Giannoulis, Christos Nousis, Maria Krokou, Ifigeneia Zikou, Ioannis Malogiannis

**Affiliations:** 1 Laboratory of Psychometrics and Neuropsychology, Eginition Hospital, Medical School of Athens, 11528 Athens, Greece; xristosnousis@hotmail.com (C.N.);; 2 Specific Sector of Personality Disorders, Eginition Hospital, Medical School of Athens, 11528 Athens, Greece; ioannis.malogiannis@gmail.com

**Keywords:** borderline personality disorder (BPD), neuropsychology, emotional dysregulation, self-harm, psychodynamic psychotherapy, neuroimaging

## Abstract

**Background:** Borderline personality disorder (BPD) is a severe psychiatric condition characterised by emotional instability, impulsivity, interpersonal dysfunction, and self-injurious behaviours. Despite growing clinical interest, the neuropsychological mechanisms underlying these symptoms are still not fully understood. This review aims to summarise findings from neuroimaging, psychophysiological, and neurodevelopmental studies in order to clarify the neurobiological and physiological basis of BPD, with a particular focus on emotional dysregulation and implications for the treatment of adolescents. **Methods:** A narrative review was conducted, integrating results from longitudinal neurodevelopmental studies, functional and structural neuroimaging research (e.g. FMRI and PET), and psychophysiological assessments (e.g., heart rate variability and cortisol reactivity). Studies were selected based on their contribution to understanding the neural correlates of BPD symptom dimensions, particularly emotion dysregulation, impulsivity, interpersonal dysfunction, and self-harm. **Results:** Findings suggest that early reductions in amygdala volume, as early as age 13 predict later BPD symptoms. Hyperactivity of the amygdala, combined with hypoactivity in the prefrontal cortex, underlies deficits in emotion regulation. Orbitofrontal abnormalities correlate with impulsivity, while disruptions in the default mode network and oxytocin signaling are related to interpersonal dysfunction. Self-injurious behaviour appears to serve a neuropsychological function in regulating emotional pain and trauma-related arousal. This is linked to disruption of the hypothalamic-pituitary-adrenal (HPA) axis and structural brain alterations. The Unified Protocol for Adolescents (UP-A) was more effective to Mentalization-Based Therapy for Adolescents (MBT-A) at reducing emotional dysregulation compared, though challenges in treating identity disturbance and relational difficulties remain. **Discussion:** The reviewed evidence suggests that BPD has its in early neurodevelopmental vulnerability and is sustained by maladaptive neurophysiological processes. Emotional dysregulation emerges as a central transdiagnostic mechanism. Self-harm may serve as a strategy for regulating emotions in response to trauma-related neural dysregulation. These findings advocate for the integration of neuroscience into psychotherapeutic practice, including the application of neuromodulation techniques and psychophysiological monitoring. **Conclusions:** A comprehensive understanding of BPD requires a neuropsychologically informed framework. Personalised treatment approaches combining pharmacotherapy, brain-based interventions, and developmentally adapted psychotherapies—particularly DBT, psychodynamic therapy, and trauma-informed care—are essential. Future research should prioritise interdisciplinary, longitudinal studies to further bridge the gap between neurobiological findings and clinical innovation.

## 1. Introduction

Borderline personality disorder (BPD) is a chronic and severe mental condition characterised by emotional instability, unstable relationships, an unstable self-image, and impulsivity [1]. It is strongly associated with self-harm and impulsivity, with approximately 10% of individuals with BPD dying by suicide (Leichsenring et al., 2024 [2]). Affecting 1–2% of the general population, BPD accounts for up to 20% of psychiatric admissions to specialised mental health units, particularly in inpatient settings and 10% of outpatient consultations [3]. This imposes significant social and economic burdens [4].

Epidemiological studies indicate a increasing incidence of BPD diagnoses, particularly among adolescents. Data from Denmark and Canada show an increase in diagnosed cases among young females, reflecting greater clinical recognition [5]. While most personality disorder research focuses on adults, BPD often emerges in adolescence, highlighting the importance of early intervention for this age group. Although interventions such as DBT-A and MBT-A have been developed and adapted for adolescents, access to and systematic implementation of age-specific interventions remains limited in many healthcare systems [6,7]. Compared to young adults and older adults, adolescents report significantly higher frequencies of BPD traits and diagnoses compared to young adults and older adults (*p* = 0.036). Specifically, identity disturbance and impulsivity are more pronounced in adolescents (*p* < 0.001). Furthermore, adolescents report higher levels of emotional (F = 15.987, *p* < 0.001) and physical abuse (F = 12.942, *p* = 0.002), whereas older adults report higher levels of emotional and physical neglect. Logistic regression analysis identifies key BPD criteria and childhood maltreatment (CM) subtypes that differentiate adolescents from adults (Li et al., 2025 [8]). These findings emphasise the importance of age-specific interventions and highlight the need for longitudinal research to improve our understanding of the developmental trajectory and causal mechanisms underlying these differences [8].

Further neurobiological findings further highlight early vulnerability, with evidence linking decreased amygdala volume in childhood to the subsequent onset of BPD symptoms, suggesting the existence of a stable neural marker [9]. There is an established association between amygdala volume in middle childhood and BPD symptoms in late adolescence. This volumetric relationship is specific to the amygdala and remains significant when accounting for major depressive disorder (MDD). These findings suggest that neurodevelopmental markers may be present relatively from an early age and remain stable over time, as amygdala intercept, rather than slope, is associated with BPD symptoms [9]. These findings are consistent with research identifying neuropsychological abnormalities in emotion regulation, impulse control, and social cognition as key contributors to BPD [10].

To address these vulnerabilities, novel interventions are being developed. In recent years there has seen an increase in digital and hybrid interventions aimed at increasing accessibility and engagement for adolescents with BPD. One such innovation is AIR therapy, a clinician-assisted e-therapy, enhances accessibility for adolescents with BPD [11]. Comparative trials indicate that the Unified Protocol for Adolescents (UP-A) is more effective than Mentalization-Based Therapy (MBT-A) in reducing emotional dysregulation, although identity and interpersonal disturbances remain difficult to treat [7]. Despite its shorter duration and lower intensity, UP-A demonstrates greater efficacy in targeting emotional dysregulationq however, both treatments show limited impact on identity and interpersonal disturbances [7]. Advances in neuromodulation and personalised treatment approaches thatintegrate conventional psychotherapy with targeted interventions, hold promise for improving outcomes [12].

Despite growing clinical recognition, BPD remains one of the most stigmatised psychiatric disorders. Continued research is needed to refine developmental models, optimise treatment strategies, and reduce stigma by improving understanding of the disorder’s neurodevelopmental and psychosocial foundations.

This review aims to:

1. review studies that explain the neuropsychological processes of BPD, particularly focusing on emotional dysregulation, impulsivity, interpersonal dysfunction and self-harm and
2. to determine the existing knowledge gaps and suggest potential future studies that will allow a more complete understanding of the disorder.

## 2. Methodology

This integrative literature review was designed using guidance adapted from the PRISMA statement and Torraco (2005) [13]. The aim was to summarise the empirical and theoretical contributions from a multidisciplinary body of literature.

### 2.1. Eligibility Criteria

The included studies were peer-reviewed and published between 2000 and 2025. Theyaddressed the neuropsychological or neurobiological dimensions of BPD. Articles thatfocused solely on pharmacological treatment without reference to the underlying neuropsychology were excluded.

### 2.2. Information Sources and Search Strategy

The literature search was conducted between January and March 2025 using four major databases: PubMed, PsycINFO, ScienceDirect, and Google Scholar. The search aimed to identify peer-reviewed articles published in English between January 2000 and March 2025. Both empirical and theoretical studies were eligiblefor inclusion if they contributed to the neuropsychological understanding of BPD.

The following Boolean search strings were employed:

“Borderline Personality Disorder” AND neuropsychology

“BPD” AND “emotional dysregulation” AND “amygdala” OR “prefrontal cortex”

“Borderline Personality Disorder” AND neuroimaging OR fMRI OR PET

“BPD” AND trauma AND HPA axis

“Impulsivity” AND “orbitofrontal cortex” AND “BPD”

“Oxytocin” AND “Borderline Personality Disorder”

“Borderline Personality Disorder” AND adolescence AND intervention

Additional studies were retrieved through backward reference searching and citation tracking of key systematic reviews.

### 2.3. Selection Process

A total of 152 articles were initially identified through database searches and manual searches. After removing duplicates (n = 40), 112 articles remained for screening. Two reviewers assessed titles and abstracts independently based on the following inclusion criteria:

- The study must address neuropsychological, neurobiological, or psychophysiological processes in BPD.
- It must be published in a peer-reviewed journal.
- Studies with adolescent, adult, or mixed samples were eligible.
- Both clinical trials and observational studies were included.
- Exclusion criteria were:
- Studies focusing solely on pharmacological outcomes without reference to underlying neuropsychology.
- Non-English publications.
- Grey literature, dissertations, or conference abstracts were also excluded.

**Figure 1:**
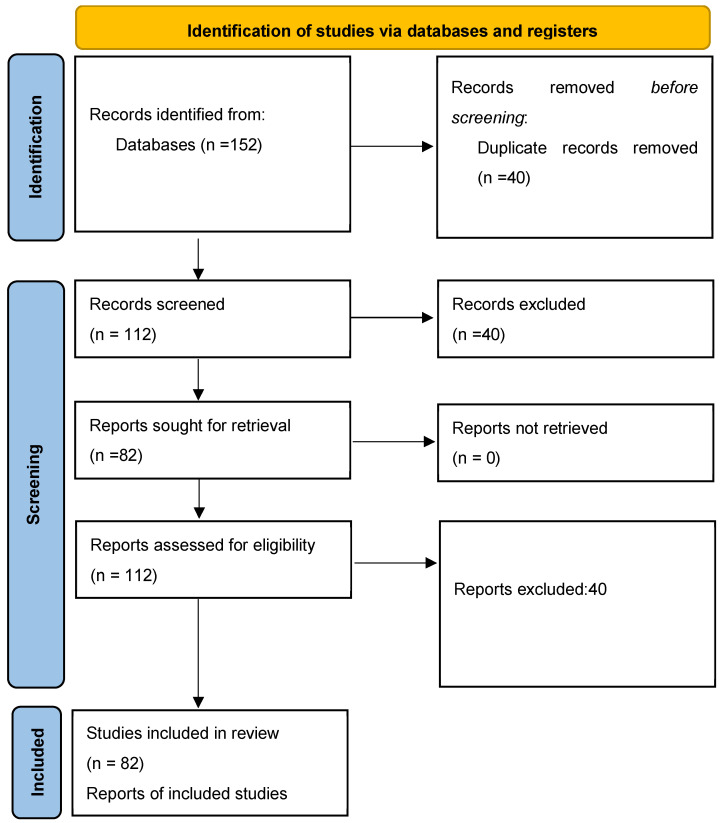
PRISMA flow diagram [14] illustrating the study selection process. A total of 152 records were initially identified through database searches. After removing duplicates, 112 records were screened. Following abstract screening, 30 records were excluded, and 82 full-text articles were assessed for eligibility. No additional exclusions were made at the full-text stage, resulting in 82 studies being included in the integrative review.

Following title and abstract screening, 82 full-text articles were included in the final synthesis. No studies were excluded at the full-text stage. A PRISMA flow diagram (Figure 1) has been added to illustrate the full screening process.

### 2.4. Data Extraction and Synthesis

Key themes were extracted manually and categorised under the following core symptom domains: emotional dysregulation, impulsivity, interpersonal dysfunction, and self-harm. Synthesis involved the cross-disciplinary integration of neuropsychological, psychodynamic, and developmental findings.

## 3. Neuropsychological Symptom Domains of BPD

### 3.1. Diagnostic Criteria

The DSM 5 classifies BPD as a personality disorder type characterised and widespread emotional instability, as well as instability in self-image, interpersonal relationships and impulsivity [1]. Criteria include emptiness, fear of abandonment, anger, and recurrent self-harming behaviours [1]. In contrast, the International Classification of Diseases, Eleventh Edition (ICD-11) has a similar categorisation of BPD, focusing on the ongoing emotional and relational difficulties, though the diagnostic criteria differ somewhat [15].

This is important because an accurate and timely diagnosis of BPD can prevent misdiagnosis and delay in the start of treatment which can worsen the disorder’s impact on the individuals and their society. The similarities between the DSM-5 and ICD-11 criteria make it easier for clinicians to use diagnostic tools across different clinical settings.

### 3.2. Core Symptoms

#### 3.2.1. Emotional Dysregulation

The main characteristic of BPD is emotional instability which is manifested by rapid mood swings and increased sensitivity to stress. These symptoms are said to be caused by increased activity of the amygdala and an increased emotional response, as well as decreased activity in the prefrontal cortex, which is the regulator of emotions [16]. This imbalance leads to the severe difficulties that BPD patients experience in the management of their emotional state.

#### 3.2.2. Impulsivity

Some of the impulsive behaviours observed in BPD include spending, substance abuse, and self-harm. These behaviours have been associated with orbitofrontal cortex dysfunction and dysregulation of serotonergic pathways affecting self-control and reasoning [17].This can make the situation worse in terms of the consequences, for example legal and financial issues.

#### 3.2.3. Interpersonal Dysfunction

People with BPD tend to experience intense anxiety about being left alone and form unstable relationships that can shift from fully positive to completely negative in a short period of time. The underlying theory of these interpersonal difficulties include attachment theory and social cognition deficits, which are related to the default mode network (DMN) [18].

#### 3.2.4. Self-Harm

Self-mutilation is a common behaviour in patients with BPD and is considered an attempt to manage emotional pain. Research indicates that self-injury triggers the endogenous opioid system, leading to pain relief and the alleviation of negative emotions, as well as promoting a sense of calm [19]. Furthermore, self-harm is also related to dysregulated dopamine which leads to the repetition of the act as it provides the individual with temporary feeling of relief or happiness [20].

### 3.3. Comorbidity

This is because BPD is often accompanied by other psychiatric disorders, which complicates the management of the condition. There is a high prevalence of comorbidity with major depressive disorder, generalised anxiety disorder, and post-traumatic stress disorder (PTSD) [21]. BPD is rooted in childhood trauma, which also increases the likelihood of a patient having PTSD. Substance use disorders affect more than half of the BPD population, worsening impulsivity and emotional regulation [22].

These co-occuring disorders highlight the importance of comprehensive management plans. Thus, treating of these co-occurring symptoms is essential for improving treatment results and preventing recurrence [23].

### 3.4. Functional Impairments

The erratic behaviour that is characteristic of BPD affects an individual’s day to day living of an individual and interferes with their social, occupational and personal functioning. Research has also indicated that people with BPD have difficulty in retaining employment, establishing sustainable relationships and achieving personal goals [4].

Due to frequent hospitalizations and visits to emergency centres, the economic impact of BPD is quite high. Preventing emotional crises and reducing self-harm behaviours can decrease these systemic costs [3] (Table 1).

## 4. Neuropsychological Foundations of BPD

### 4.1. Emotional Dysregulation

The defining feature of BPD is emotional dysregulation, which is always accompanied by neural abnormalities. The amygdala, a structure whose activity is elevated in BPD, processes emotional stimuli, while the prefrontal cortex, whose activity is diminished in BPD, executes top-down emotion regulation [24,26]. This imbalance is responsible for the dramatic mood swings and the difficulty in regulating emotions which are characteristic of individuals with BPD.

Altered serotonin pathways have been linked to the increased emotional responsiveness observed in BPD patients. St. Serotonin transporter availability is associated with impulsive and aggressive behaviour, while disruptions to the dopamine system lead to emotional intensity and transient paranoia induced by stress [26]. These neurochemical findings demonstrate the complex interaction between systems and neurotransmitter emotional dysregulation.

### 4.2. Impulsivity and Behavioral Dyscontrol

Impulsivity in BPD is often associated with deficits in cognitive executive function, including inhibitory control, decision-making, and working memory. Functional imaging studies show that the dorsolateral prefrontal cortex is less active in people with BPD, leading to deficits in cognitive control and increasing the likelihood of impulsive actions [17].

Abnormalities in the orbitofrontal cortex, which is involved in reward processing and impulse control, also increase impulsivity in BPD [27]. These deficits prevent the individual from considering the long-term consequences in the long run and may lead to impulsive and poor decision-making.

A tendency towards elevated impulsivity and risk-taking behaviours in adolescents diagnosed with BPD can result in negative health consequences, such as suicide and substance abuse [28]. Furthermore, individuals who receive an early diagnosis of BPD may experience greater disorder severity and a less favourable prognosis [29].

### 4.3. Interpersonal Dysfunction

The neural circuits of social cognition are also affected in BPD, manifesting as interpersonal difficulties such as a fear of abandonment and unstable relationships. The default mode network (DMN), which is involved in self-related processing and social processing, is also altered in BPD. This leads to increased sensitivity to rejection and poor trust [18].

The oxytocin system, which facilitates bonding and trust, is also dysregulated in people with BPD who self- injure. Low oxytocin levels can exacerbate social stress, making individuals more susceptible to interpersonal conflict [30].This biological understanding of the disorder aligns with clinical observations of unstable relationships in patients with BPD.

### 4.4. Self-Harm

Self-harm, also known as non-suicidal self-injury (NSSI), is a common behaviour among people with BPD. Although it typically lacks suicidal intent, it is a serious clinical issue that significantly increases the risk of suicide and exacerbates both emotional and physical distress. In BPD, self-harm often serves as a maladaptive mechanism for regulating overwhelming emotional states. It is essential to understand the neuropsychological factors underlying self-injurious behaviours, such as dysregulated affect, altered pain perception, and deficits in impulse control, in order to identify the roots of these behaviours and guide effective treatment approaches [31] (Table 2).

### 4.5. Pain Modulation and Reward Mechanisms

Another interesting feature of self-harm is that it can provide emotional comfort through pain. This is partly due to the endogenous opioid system, which produces natural pain relievers that help in the patient to feel calm [25]. Some people with BPD feel painfultly depressed for most of their lives and thus may turn to self-harm as a way of feeling something other than pain, if only temporarily..

Self-harm also affects the brain’s reward system the dopaminergic pathways. The elation or relief experienced after self-harm is linked to increased dopamine levels in the brain, which create reward- like activities in the nervous system [20]. This reinforcement schedule also explains why the behaviour can become compulsive, despite its adverse long-term effects..

### 4.6. Emotional Dysregulation and Neural Dysfunctions

The dysregulation of emotions is exacerbated by the neural imbalance that is characterised by an overactive amygdala and an underactive prefrontal cortex in BPD. This imbalance leaves the patients in frequent emotional pain, which may lead them to engage in self-injurious behaviors to alleviate it [16].

The serotonin system, which is involved in regulating of mood and impulsive behaviour, is often defective in BPD. Stimulating of the serotonin system has been associated with reduced aggression and impulsive behaviours, particularly during periods of emotional overload [32].

### 4.7. Trauma, Stress Response, and Neural Plasticity

It is important to note that most individuals with BPD individuals have experienced trauma in the past which affects the HPA axis. This leads to the cortisol levels in the body flaring up, and therefore, the patient experiences more emotional pain and may resort to self-harm [33].

Trauma affects brain areas such as the hippocampus and prefrontal cortex, which are linked to emotion regulation and executive functions. These structural changes increase the risk of self-harm especially during periods of stress [34].

### 4.8. Oxytocin and Social Neuropsychology

The oxytocin system, which plays a role in social bonding and trust, is also dysregulated in individuals with BPD who engage in self-harm. Lower levels of oxytocin increase anxiety and feelings of rejection and loneliness, which may lead to self-injurious behaviours in response to interpersonal stress [35].

### 4.9. Behavioral Reinforcement and Habit Formation

Aggression is said to act as a negatively reinforcing behaviour. The relief that the patient feels after self-harm herself/himself and the desire to feel that way again are the factors that may lead to the repetition of the behaviour when the patient is emotionally overwhelmed [20].

Self-harm behaviors when practiced over a period of time create strong connections in the network of the brain that leads to negative coping mechanism that are difficult to remove. Over the years, these pathways become stronger than the other, health oriented ones and thus become a source of self-injurious behaviours [25].

Thematic synthesis prioritised findings from high-quality studies, particularly those supported by longitudinal neuroimaging or robust psychophysiological designs. Themes primarily drawn from smaller or cross-sectional studies were included, but were interpreted with caution. This weighting approach is reflected in our narrative structure and is further supported by the quality appraisal provided in Supplementary Table S1.

## 5. Treatment Considerations

### 5.1. Pharmacotherapy

Medications that include SSRIs and dopamine modulators to manage serotonergic and dopaminergic systems may help to decrease impulsive behaviours and emotional responsiveness. Naltrexone has been shown to play a role in blocking the rewarding effect of self-harm by manipulating of the endogenous opioid system [36].

### 5.2. Neuromodulation Techniques

Novel neuromodulation strategies such as transcranial magnetic stimulation (TMS) and neurofeedback, have the potential to normalise the neural networks that are involved in emotion regulation and impulsive behaviours [37].

### 5.3. Psychotherapeutic Interventions

Psychotherapy remains essential in the management of self-harm in BPD.

DBT: In Linehan’s (1993) DBT, the emphasis is on teaching distress tolerance and emotional regulation skills to prevent self-harm and reduce its frequency and severity [38].

Trauma Informed Approaches: EMDR and somatic therapies address trauma-related dysregulation, a common precursor to self-harming behaviours [39].

## 6. Advances in Neuroimaging and Psychophysiological Research

Neuroimaging analysis using fMRI has revealed increased activity in the limbic system, particularly in the amygdala when processing emotional stimuli in individuals with BPD [26,40]. A lack of integration between the amygdala and the medial prefrontal cortex has also been observed, indicating the poor control over emotions that leads to mood swings and impulsive behaviours [26].

## 7. Neurodevelopmental Perspectives

BPD is shaped by the complex interplay of genetic predisposition and early-life environmental adversity. Neurodevelopmental research highlights how childhood trauma, disrupted attachment, and early stress can alter neural circuits associated with emotion regulation and impulse control.

### 7.1. Early-Life Adversity and the Brain

Approximately 75% of individuals with BPD report experiencing childhood trauma [41]. Such experiences can alter the hypothalamic-pituitary-adrenal (HPA) axis, thereby increasing cortisol reactivity and inducing structural changes in the hippocampus and prefrontal cortex [33,34].

### 7.2. Developmental Trajectories

The adolescent brain undergoes heightened plasticity, making it especially vulnerable to stress. Prolonged amygdala hyperactivation, paired with delayed maturation of prefrontal regulatory regions, may establish a trajectory towards emotional dysregulation and impulsivity [42].

### 7.3. Gene–Environment Interactions

Twin studies suggest that BPD traits are moderate heritability (at around 40%) of BPD traits, but epigenetic mechanisms mediate how adverse experiences activate genetic vulnerabilities [43,44]. For example, 5-HTTLPR polymorphisms may increase susceptibility to negative and positive environmental influences [45].

### 7.4. Clinical Implications

These findings emphasise the importance of preventive strategies such as trauma-informed parenting education, the early detection of at-risk youth through neurodevelopmental screening, and the early engagement of therapy using DBT-A or MBT-A.

## 8. Implications for Clinical Practice

Treating BPD requires integrative, multimodal strategies that address both neurobiological deficits and relational patterns. Recent innovations in psychotherapy, pharmacotherapy, and neuromodulation (see Table 3) have expanded the treatment landscape. However, these approaches should be tailored to specific symptoms and developmental stage of the individual.

### 8.1. Pharmacological Approaches

Although no medication is curative, SSRIs and mood stabilisers may reduce affective instability and impulsivity. Naltrexone has shown promise in breaking the reinforcing cycle of self-harm by modulating of the endogenous opioid system [36].

### 8.2. Neuromodulation

TMS targeting the dorsolateral prefrontal cortex has demonstrated preliminary efficacy in improving emotion regulation [46] while neurofeedback may promote cognitive control in high-reactivity states [47].

**Table 3:** Overview of treatment modalities for Borderline Personality Disorder (BPD), organized by primary symptom domain targeted, strength of empirical evidence, and representative references. This table aligns therapeutic interventions with specific neuropsychological dysfunctions to guide clinical decision-making and research development.

Treatment Modality	Primary Symptom Domain	Evidence Strength	Key References
**1. Dialectical Behavior Therapy (DBT)**	Emotional dysregulation, self-harm	Strong	[38,48]
**2. Unified Protocol for Adolescents (UP-A)**	Emotional dysregulation	Moderate	[7]
**3. Mentalization-Based Therapy (MBT)**	Mentalization deficits, interpersonal dysfunction	Moderate	[49]
**4. Transference-Focused Psychotherapy (TFP)**	Attachment-related affect regulation	Emerging	[50]
**5. EMDR (Eye Movement Desensitization and Reprocessing)**	Trauma-linked emotional dysregulation	Emerging	[51]
**6. Pharmacotherapy (SSRIs, mood stabilizers, naltrexone)**	Affective instability, impulsivity	Moderate	[36,52]
**7. Neuromodulation (TMS, Neurofeedback)**	Impaired prefrontal regulation	Emerging	[46,47]

A developmental-neurobiological perspective can help us to better understand the differential efficacy of the Unified Protocol for Adolescents (UP-A) mk Mentalization-Based Therapy for Adolescents (MBT-A). UP-A focuses on transdiagnostic emotional regulation strategies that directly target neurocognitive deficits in prefrontal control and amygdala hyperreactivity, which are neurological patterns commonly observed during adolescence in individuals at risk of BPD. In contrast, MBT-A emphasises the development of mentalising capacity and secure attachment representations, which may require a higher level of developmental maturity and therapeutic engagement over time. UP-A’s comparatively greater short-term efficacy in reducing emotional dysregulation [7] may thus reflect its alignment with dominant neurobiological vulnerabilities of adolescents, while MBT-A may be more effective in addressing interpersonal dysfunctions that stabilise later in development.

## 9. Future Research Directions

Improving our understanding of and ability to manage BPD requires more innovative research approaches that combine neuroscience, psychophysiology and clinical practice. The following are key areas of further research could help improve therapeutic interventions and understanding of the disorder [2].

### 9.1. New Technologies in Neuropsychology

New advances in neuroimaging such as high-resolution MRI and diffusion tensor imaging (DTI) present new possibilities for exploring structural and functional connections in the BPD population. These tools can help in understanding the changes in networks like the default mode network (DMN) and the amygdala-prefrontal cortex circuit [53].

Biomarkers for BPD can be identified through imaging, genetic and psychophysiological assessments and this can change the current diagnostic processes and treatment monitoring. For instance, amygdala-prefrontal connectivity metrics and heart rate variability (HRV) can be used as objective markers of emotional regulation and stress response. It is possible to use machine learning algorithms to combine multiple datasets to identify treatment response and BPD subtypes based on neuropsychological data. Such methods can assist in management of treatment and improve the accuracy of the therapeutic process [54].

### 9.2. Personalized Treatment Approaches

Future work should be directed towards the formulation of more tailored treatment plans based on the individual’s neuropsychological assessment. Thus, clinicians can understand the specific deficits, for instance, in amygdala activity or in the activity of the prefrontal cortex, to which they can direct the treatment [53,54]. Genetic and epigenetic information can also be used in clinical practice and may help in individualizing treatment. For example, patients with polymorphisms of the serotonin transporter gene may benefit from serotonin-targeted interventions, while epigenetic markers may help in selecting trauma-informed therapies [55].

### 9.3. Longitudinal Studies

Longitudinal studies are crucial for understanding the course of BPD in the life span. Such studies may help in understanding how the neuropsychological features like emotional dysregulation and impulsivity change over time and how the interventions affect these changes [4]. Thus, the development of the phenotype can help in the determination of the critical periods during which the given intervention may be most effective. For instance, adolescence is a stage of increased plasticity and symptom development, thus it is an important period for prevention and early intervention [42].

### 9.4. Increasing Trauma Research

More work is needed to determine how trauma influences the development of the brain and increases the risk of BPD. The understanding of the relationship between HPA axis, limbic system, and prefrontal cortex in trauma victims can assist in the development of trauma informed care [33].The impact of different trauma-informed therapies like EMDR and somatic experiencing on neural and psychological functioning may be useful in the improvement of trauma treatment for BPD patients [51].

### 9.5. Interdisciplinary and Cross-Cultural Research

Future work should incorporate a interdisciplinary approach that includes the participation of neuroscientists, clinicians and social scientists. This approach can provide a biopsychosocial perspective, which can help in the comprehensive understanding of BPD. BPD may present differently and be managed differently across different cultures. Comparative studies can help in understanding how cultural factors, such as norms, stigma, and healthcare systems affect the expression and care of BPD and may provide information on culturally appropriate interventions [56].

### 9.6. Innovation in Therapeutic Modalities

The increasing popularity of telehealth and application-based interventions for BPD present new opportunities for development. Recent literature highlights the effectiveness of digital DBT modules in enhancing emotion regulation, a core target in BPD treatment. For instance, Jadhakhan et al. (2022) [57] demonstrated that digital interventions—such as mobile apps and web-based platforms—can improve emotional regulation capacities and accessibility to care. These findings support the expansion of clinician-guided and self-paced digital DBT formats as viable teletherapy models for BPD [57].

### 9.7. Advocacy and Stigma Reduction

Despite advances in the clinical understanding of Borderline Personality Disorder (BPD), stigma remains a major barrier to diagnosis, treatment, and recovery. Research consistently shows that BPD is one of the most stigmatized psychiatric diagnoses, even among mental health professionals [58,59].Stigmatizing attitudes may lead to underdiagnosis, diagnostic overshadowing, therapeutic nihilism, or outright exclusion from services—particularly in emergency or primary care settings. These negative perceptions contribute to fragmented care, diminished treatment engagement, and internalized stigma among individuals with BPD, further exacerbating symptoms and social withdrawal.

Efforts to improve public and professional awareness must therefore emphasize the neurobiological and developmental underpinnings of BPD, countering the misconception that it is a purely volitional or untreatable disorder. Training initiatives focused on trauma-informed care, emotion dysregulation, and neurodevelopmental vulnerability may help reframe BPD as a valid and treatable condition.

To guide future research and intervention design, the following questions are proposed:

1. What are the effects of clinician-focused anti-stigma interventions on diagnostic accuracy and treatment engagement for BPD?

(e.g., pre/post training designs in psychiatric and primary care settings)

2. How does public stigma around BPD compare to that of other psychiatric disorders, and what factors mediate these attitudes?

(e.g., comparative cross-sectional surveys with regression modeling)

3. Can digital or narrative-based campaigns (e.g., patient storytelling, VR empathy training) reduce stigma and improve attitudes toward individuals with BPD among the general public and healthcare trainees?

(e.g., randomized controlled trials or longitudinal evaluation of outreach programs)

Addressing stigma through targeted educational and systemic strategies is essential not only for improving treatment access but also for enhancing the quality of therapeutic relationships and long-term outcomes.

### 9.8. Integration of Neuroscience and Psychotherapy

Future work can also involve the relationship between psychotherapy and neural plasticity and how therapies like DBT, TFP and Psychoanalysis affect the brain activity and connectivity in the long run. These insights could help in the improvement of the therapeutic techniques to the brain function.

### 9.9. Psychoanalysis and Neuropsychology in BPD

However, the following are some of the areas where psychoanalysis and neuropsychology have provided valuable insights in the management of BPD:

#### 9.9.1. Emotional Dysregulation and Early Object Relations

Psychoanalysis: Emotional dysregulation in BPD is a consequence of failed attachment and object relations in early relationships [60]. Splitting and projective identification are most evident when individuals have fluctuating attitudes toward others and themselves, which is in congruence with their intrapsychic processes [61]. Lack of mentalization as defined by Fonagy et al. (2002) only adds to the emotional chaos [62].

Neuropsychology: Emotional dysregulation is associated with increased activity in the amygdala and decreased activity in the prefrontal cortex [26] which makes for impaired top-down regulation of emotions. Analysts argue that such findings show that the patient has extremely heightened and unmanageable affect as a result of faulty early caregiving [18].

#### 9.9.2. Impulsivity and the Ego’s Structural Weakness

Psychoanalysis: Impulsivity in BPD is due to the poor regulation of the id by the ego in relation to reality [63]. According to Kernberg (1985), this is attributed to poor ego development and integration of aggression and libido [61].

Neuropsychology: Impulsivity is related to orbitofrontal cortex lesions and to cognitive executive functions [17]. Psychoanalysis sees this as the failure of the internalized representational processes, which are a consequence of developmental trauma and lack of mirroring in early childhood according to Winnicott (1960) [64].

#### 9.9.3. Interpersonal Dysfunction and Attachment Theory

Psychoanalysis: Unpredictable relationships in BPD are a result of unstable dependency needs and fear of abandonment as postulated by attachment theory [65]. According to Winnicott (1965) the “holding environment” is the importance of early caregiving in creating a coherent self which is broken in BPD [66].

Neuropsychology: Interpersonal dysfunction is linked with abnormalities in the default mode network (DMN), which is involved in self and other related processing [67]. Oxytocin signaling has also been implicated as a biological marker of attachment and related relational instability [68].

#### 9.9.4. Self-Injury and Primitive Defense Mechanisms

Psychoanalysis: Self injury is seen as a form of projective identification, where the individual releases unbearable affect through physical action [60]. Freud’s (2015) concept of the death drive (Thanatos) can be used to explain the cyclical and self-destructive nature of self-injury [69].

Neuropsychology: Altered pain perception and the HPA axis dysregulation are some of the neurological features of self-injury [70]. According to psychoanalysis these somatic manifestations are a proof of trauma and unprocessed affect [71].

#### 9.9.5. Trauma, Regression and the HPA Axis

Psychoanalysis: Trauma leads to regression to more basic forms of functioning which cannot be symbolized. The concept of beta elements that are raw and cannot be easily processed helps in the understanding of the fragmentation observed in BPD [72].

Neuropsychology: Trauma related structural changes in hippocampus and prefrontal cortex is well documented [73]. These changes are in line with the psychoanalytic concept of developmental assemblages which are thought to be caused by traumatic early relational experiences [49].

#### 9.9.6. Perspectives Integration

##### Mentalizing and Prefrontal Cognitive Functions

Mentalizing Based Therapy (MBT) works on the issues of reflective functioning and the connection between the prefrontal cortex and the limbic system [74].

##### Trauma and Neural Plasticity

Both psychoanalysis and neuropsychology acknowledge that neural and psychological repair is possible through therapeutic process. The progress in neuroimaging has shown that the individuals who have been treated have better connectivity [48].

#### 9.9.7. Symbolization and Neural Integration

The conversion of beta elements into alpha elements in the course of therapy [72] is consistent with the neuroplasticity observed in the course of psychotherapeutic treatments, and the integration of emotional and cognitive processes [75].

## 10. Discussion

Borderline Personality Disorder (BPD) is a psychiatric disorder that is characterized by emotional instability, impulsive behavior, relationship problems, and self-harming behaviors. The study is attempting to identify the neuropsychological processes of BPD symptomatology through the integration of neuroimaging studies, psychophysiological assessments, and neurodevelopmental evaluations.

Literature review encompassed neuroimaging research combined with psychophysiological examinations and neurodevelopment studies. The purpose of this study was to determine what neural circuits and physiological processes are involved in emotional dysregulation, impulsivity, interpersonal dysfunction, and self-injurious behavior. Research indicated that decreased amygdala volume at the age of 13 or younger can be utilized as an early marker for the development of BPD, and longitudinal research demonstrated a static correlation between amygdala volume in childhood and later BPD symptoms. This neurodevelopmental vulnerability is in line with findings that adolescents exhibit significantly higher levels of BPD symptom frequencies, namely identity disturbance and impulsivity, and higher exposure to childhood maltreatment, with abuse being more common in youths and neglect being more common in adults.

Neuroimaging findings illustrate that the amygdala is hyperactive while the prefrontal cortex is hypoactive in patients with BPD, which accounts for their defective emotion regulation. The orbitofrontal cortex is abnormal, with executive function impairments being related to impulsivity. Interpersonal dysfunction is associated with the breakdown of the default mode network and oxytocin signaling. Self-injury is a neuropsychological mechanism that provokes emotional release and pain control following traumatic injury to modify the HPA axis and produce structural changes to the hippocampus and prefrontal cortex. Neuromodulation interventions, when combined with psychophysiological metrics like heart rate variability, yield measurable results for emotional and stress management abilities. Treatment methods are being developed to reverse these deficits, with studies demonstrating the superiority of the Unified Protocol for Adolescents (UP-A) over Mentalization-Based Therapy (MBT-A) in decreasing emotional dysregulation; however, challenges persist in the treatment of identity and interpersonal pathology. Although briefer in duration and intensity, UP-A demonstrates more favorable results in the treatment of emotional dysregulation than MBT-A, thereby calling for the development of customized interventions for adolescent populations.

### 10.1. Digital Innovations in DBT and Emotion Regulation

The emergence of teletherapy and digital interventions has expanded access to evidence-based treatments for BPD, particularly among adolescents and underserved populations. Digital adaptations of Dialectical Behavior Therapy (DBT), including mobile apps and web-based modules, have shown promise in enhancing emotion regulation—a central deficit in BPD. Jadhakhan et al. (2022) reviewed digital technologies targeting emotion regulation and concluded that such platforms can effectively support skill acquisition and therapeutic engagement [57]. Importantly, the use of Virtual Reality (VR) as a training environment for emotion regulation skills has shown early promise in related psychiatric populations, serving as a proof of concept for immersive, experiential learning. These findings align with the neurobiological emphasis on early intervention and support the integration of digital DBT formats within stepped-care models, especially when in-person therapy is not feasible. However, further research is required to evaluate long-term outcomes, therapeutic alliance in digital contexts, and the neurocognitive impact of remote interventions.

The conclusions drawn in this review give greater interpretive weight to findings from studies rated as high quality based on our CASP-based appraisal. This includes studies with robust methodological design, clear aims, and validated outcome measures. Conversely, theoretical and preliminary findings—while informative—were integrated more cautiusly and framed as areas requiring further empirical validation.

### 10.2. Current Gaps and Controversies

Despite growing clarity on the neurobiological underpinnings of BPD, several areas of contention remain. First, the role of oxytocin in modulating interpersonal functioning is inconsistent across studies, with some reporting prosocial effects and others noting paradoxical increases in mistrust or social anxiety. These discrepancies may be influenced by trauma history, attachment style, or genetic variation in oxytocin receptor expression. Second, while DBT remains the gold standard for reducing emotional dysregulation, comparative trials of newer interventions—such as UP-A versus MBT-A—suggest differential efficacy across domains (emotional vs. relational), yet head-to-head studies remain limited. Third, although neuromodulation and digital therapies show promise, evidence remains preliminary, and their long-term impact on neural plasticity and behavior is not well established. Finally, psychodynamic formulations provide a rich clinical framework, but more empirical work is needed to link these theories to specific neurobiological correlates. These gaps highlight the need for integrative, longitudinal studies that combine clinical outcomes with neurobiological and genetic data.

This summary reflects on the different neuropsychological factors that are associated with BPD, including emotional regulation, impulsivity, relationships, and self-mutilation.

The evidence presented demonstrates that the amygdala is functioning at a heightened level, and the prefrontal cortex is less functional, along with disruptions in the regulation of serotonin and dopamine, and implications for other neural systems, including the default mode network (DMN). Recent neuroimaging and psychophysiological studies have been capable of explaining the structural and functional changes observed in BPD, and development in the understanding of trauma-related neurodevelopment has been beneficial in furthering the understanding of the disorder′s etiology.

The conclusions of this review suggest that therapeutic models must include neuropsychological information. Individualized treatment protocols, based on neuroimaging and psychophysiological assessment, can improve treatment outcomes.

New treatment methods involve neuromodulation and pharmacologic intervention that can attempt to remediate particular neural system deficits, hopefully enhancing emotional regulation and avoiding impulsive behavior. Psychotherapies like Dialectical Behavior Therapy (DBT), Transference-Focused Psychotherapy (TFP), Bion′s theory, Mentalization-Based Therapy (MBT), and Trauma-Focused Cognitive Behavioral Therapy (TF-CBT) are still at the core of treatment for Borderline Personality Disorder (BPD). These approaches are consistent with neurobiological research and emphasize the need for incorporating psychodynamic principles in neuroscience-informed treatments. This synthesis underscores the need for greater interdisciplinary research and longitudinal investigations. Emerging technologies, including machine learning and neuroimaging, have the potential to enhance diagnostic precision and render treatment planning more personalized. Research in the future must fill gaps in trauma studies and investigate the influence of cultural and system variables on BPD treatment. Decreasing BPD stigma is critical to enhancing access to healthcare services and patient outcomes.

Public and professional awareness efforts must focus on the neurobiological underpinnings of BPD, stressing that it results from comprehensible and treatable causes. The intricacies of BPD call for the integration of efforts among neuroscientists, clinicians, psychotherapists, and policymakers to enhance care strategies. The gap between theory and practice can only be filled through concerted efforts to enable proper translation of scientific information into clinical practice in everyday life. Elucidating the BPD neurodevelopmental pathways highlights the need for early detection and intervention, especially in vulnerable children and adolescents. Early intervention may reverse or weaken the disorder′s long-term effects and enhance the quality of life for those affected. The role of newer treatment modalities such as neuromodulation and digital interventions in the management of BPD is still evolving. The goal is to develop a hybrid model that merges new technologies with traditional psychotherapeutic approaches to deliver a more adaptive and personalized treatment plan. Lastly, BPD is an extremely challenging and complex disorder that requires a multifaceted, evidence-based approach. By the integration of neuropsychological assessment, interdisciplinary teamwork, and creative interventions, practice can be pushed toward whole-person, patient-centered care for individuals with BPD. Continued research, advocacy, and the development of clinical practice remain essential to working with those who live with this long-term illness.

### 10.3. Limitations

While this review integrates diverse sources to provide a comprehensive neuropsychological framework for understanding BPD, several limitations should be acknowledged. First, due to its narrative and integrative nature, a formal risk-of-bias assessment (e.g., Cochrane Risk of Bias Tool) was not applied. Instead, studies were qualitatively evaluated based on design quality, sample size, and clinical relevance. This subjective appraisal may introduce variability and reduce replicability. Second, although care was taken to include high-impact studies across modalities, the absence of a meta-analytic component limits the ability to assess effect sizes or statistical heterogeneity. Finally, publication bias and language bias may have influenced the scope of included literature, as only English-language peer-reviewed publications were considered.

## 11. Conclusions

This integrative review has outlined the neuropsychological mechanisms underlying BPD and their clinical relevance. By synthesizing evidence from neuroimaging, psychophysiological research, developmental trauma studies, and therapeutic approaches, the review underscores the importance of an interdisciplinary treatment model. Future research must bridge neurobiology with clinical innovation to create personalized, culturally sensitive, and developmentally attuned interventions. Enhanced public and professional awareness of the neuropsychological foundations of BPD may also reduce stigma and improve care.

## Figures and Tables

**Table 1:** Core symptom domains in Borderline Personality Disorder (BPD), associated neural correlates, and representative findings. Emotional dysregulation, impulsivity, interpersonal dysfunction, and self-harm are each linked to distinct patterns of brain activity and neurochemical processes. The table summarizes how specific brain systems contribute to characteristic symptoms of BPD and cites key studies supporting these associations.

Domain	Neural Correlate	Key Findings	References
Emotional Dysregulation	Amygdala hyperactivity, PFC hypoactivity	Reduced regulation capacity, high reactivity	Silbersweig et al., 2007 [24]
Impulsivity	OFC and DLPFC dysfunction	Poor inhibition and decision-making	Sebastian et al., 2014 [17]
Interpersonal Dysfunction	DMN alterations, Oxytocin signaling	Rejection sensitivity, unstable attachments	Preißler et al., 2010 [18]
Self-Harm	Opioid and dopamine system activation	Pain relief, compulsive repetition	Zhang et al., 2025 [25]

**Table 2:** Summary of core symptom domains in Borderline Personality Disorder (BPD), their associated neural correlates, and representative key studies. This table synthesizes findings across emotional, cognitive, and interpersonal domains, linking each to specific neurobiological mechanisms that contribute to BPD symptomatology.

Domain	Neural Correlates	Key Studies
**1. Emotional Dysregulation**	Amygdala hyperactivity, Prefrontal cortex hypoactivity	[16,24]
**2. Impulsivity**	Orbitofrontal cortex dysfunction, Dorsolateral PFC hypoactivity	[17,27]
**3. Interpersonal Dysfunction**	Default Mode Network (DMN) alterations, Oxytocin dysregulation	[18,30]
**4. Self-Harm**	HPA axis dysregulation, Endogenous opioid/dopamine system	[20,25]

## Supplementary Materials

The following supporting information can be downloaded at: https://www.mdpi.com/article/10.3390/jcm14144973/s1, Table S1. Quality Appraisal and Thematic Contribution of Included Studies.

## Abbreviations

BPD	Borderline Personality Disorder
APA	American Psychiatric Association
DSM-5	Diagnostic and Statistical Manual of Mental Disorders, Fifth Edition
ICD-11	International Classification of Diseases, 11th Revision
CM	Childhood Maltreatment
MDD	Major Depressive Disorder
DMN	Default Mode Network
NSSI	Non-Suicidal Self-Injury
HPA axis	Hypothalamic–Pituitary–Adrenal Axis
UP-A	Unified Protocol for Adolescents
MBT-A	Mentalization-Based Therapy for Adolescents
DBT	Dialectical Behavior Therapy
TFP	Transference-Focused Psychotherapy
MBT	Mentalization-Based Therapy
EMDR	Eye Movement Desensitization and Reprocessing
TF-CBT	Trauma-Focused Cognitive Behavioral Therapy
SSRIs	Selective Serotonin Reuptake Inhibitors
TMS	Transcranial Magnetic Stimulation
tDCS	Transcranial Direct Current Stimulation
fMRI	Functional Magnetic Resonance Imaging
DTI	Diffusion Tensor Imaging
HRV	Heart Rate Variability
5-HTTLPR	Serotonin-Transporter-Linked Polymorphic Region
DNA	Deoxyribonucleic Acid
