# Supplementary material for: Wired for Intensity: The Neuropsychological Dynamics of Borderline Personality Disorders—An Integrative Review
Source: J Clin Med. 2025 Jul 14;14(14):4973. doi: 10.3390/jcm14144973 (PMC12295329; doi:10.3390/jcm14144973)
Supplement: Supplementary file 1 [file jcm-14-04973-s001.zip › jcm-3692988-supplementary.pdf]

Study	First Author, Year	Design Type	Sample Size	Thematic Focus	Quality Appraisal	Weight in Synthesis
[9] Constantino-Pettit et al., 2025	Longitudinal neuroimaging	>200 adolescents	Amygdala development & BPD onset	High	Primary; foundational for developmental vulnerability	Primary
[16] Schmahl & Bremner, 2006	fMRI study	Moderate (n = 40–80)	Emotion regulation (amygdala/PFC)	High	Core neurobiological mechanism	Primary
[17] Sebastian et al., 2013	Cross-sectional fMRI	Moderate	Impulsivity, orbitofrontal dysfunction	High	Strong support for impulsivity–neurocircuitry link	Primary
[62] Fonagy et al., 2002	Theoretical + empirical	Review + supporting trials	Interpersonal dysfunction, DMN	Moderate	Supporting; integrated with caution	Supporting
[20] Klonsky, 2007	Narrative review	N/A	Self-harm functions	Moderate	Secondary; theoretical contribution	Contextual
[24] Silbersweig et al., 2007	Experimental fMRI	Small (n = ~30)	Emotion regulation, frontolimbic inhibition	High	Strong empirical evidence	Primary
[40] Schulze et al., 2016	Meta-analysis (fMRI)	Large	Limbic overactivity, neural imbalance	High	Confirmatory; robust synthesis	Primary
[27] Berlin et al., 2005	Functional imaging	Small	Impulsivity, OFC lesions	Moderate	Supplementary; interpreted with caution	Supporting
[35] Pierrehumbert et al., 2010	Psychophysiology	Medium	Oxytocin & interpersonal stress	Moderate	Context-dependent; included with caveats	Contextual
[32] Seo et al., 2008	Neurochemical review	N/A	Serotonin/dopamine & aggression	Moderate	Theoretical; linked to emotion dysregulation	Supporting
[34] Teicher et al., 2004	Neurodevelopmental MRI	High	Trauma-induced hippocampal alterations	High	Primary; supports trauma-related vulnerability	Primary

Study	First Author, Year	Design Type	Sample Size	Thematic Focus	Quality Appraisal	Weight in Synthesis
[42] Crone & Dahl, 2012	Developmental neuroscience	Review	Adolescent brain plasticity	High	Integrative for developmental sensitivity	Supporting

Supplementary Table S1. Quality Appraisal and Thematic Contribution of Included Studies

Appraisal Criteria:

Adapted from CASP (Critical Appraisal Skills Programme), quality was rated as:

- High: Robust methodology, clear aims, large or longitudinal samples, validated tools.
- Moderate: Acceptable design, smaller samples or cross-sectional nature, some limitations.
- Low: Not included in primary synthesis.

Weight in Synthesis:

- Primary: Directly informs core arguments and conclusions.
- Supporting: Used to triangulate or extend primary evidence.
- Contextual: Included with interpretive caution; used to frame questions or theoretical background.
